# Multi‐institutional validation of hypersight CBCT‐based dose calculation on O‐ring linacs

**DOI:** 10.1002/acm2.70512

**Published:** 2026-02-16

**Authors:** Chih‐Yuan Lin, Yi‐Ling Chen, Chia‐Chi Chang, Yin‐Hsun Hu, Chia‐Peng Pan, Fang‐Hui Liu, Hsiang‐Ping Chao, Yang‐Wei Hsieh, Yu‐Wei Lin, Chi‐Yuan Yeh, Tzu‐Yuan Chao, Shih‐Ming Hsu

**Affiliations:** ^1^ Varian Medical Systems A Siemens Healthineers Company Taipei Taiwan; ^2^ Department of Biomedical Imaging and Radiological Sciences National Yang Ming Chiao Tung University Taipei Taiwan; ^3^ Medical Physics and Radiation Measurements Laboratory National Yang Ming Chiao Tung University Taipei Taiwan; ^4^ Department of Radiation Oncology Kaohsiung Veterans General Hospital Kaohsiung Taiwan; ^5^ Department of Radiation Oncology Tungs' Taichung MetroHarbor Hospital Taichung Taiwan; ^6^ Department of Radiation Oncolongy Kaohsiung Show Chwan Memorial Hospital Kaohsiung Taiwan

**Keywords:** dose calculation, dosimetric validation, HU‐to‐density calibration curve, hypersight CBCT, multi‐institutional study, O‐Ring Linac

## Abstract

**Background:**

Conventional cone‐beam computed tomography (CBCT) systems are limited by suboptimal image quality, inaccurate Hounsfield unit (HU) calibration, and reduced reliability for dose calculation. HyperSight CBCT on the Halcyon platform offers improved HU accuracy, expanded field‐of‐view (FOV), and enhanced image quality.

**Purpose:**

This study aimed to assess the dosimetric accuracy of treatment planning using HyperSight CBCT through phantom‐based dose verification.

**Methods:**

This study included three institutions equipped with the HyperSight imaging system on the Halcyon platform, with all procedures performed after acceptance testing and calibration. Each institution generated HU‐to‐density calibration curves using computed tomography (CT) scanners and standardized phantoms, and corresponding CBCT for planning (CBCTp) scans were also acquired. Additional CBCTp scans were acquired using a consistent phantom model (062 M) across the three institutions. Reference treatment plans were created on CT images and transferred to CBCTp and CBCT datasets for dose recalculation using identical parameters. Dosimetric assessment included gamma analysis and comparisons of DVH‐based dosimetric metrics for relevant regions of interest (ROIs). End‐to‐end testing with an anthropomorphic phantom was performed using ion chamber measurements and film dosimetry at brain, bone, and thorax locations.

**Results:**

HU‐to‐density curves showed consistent behavior across institutions, with larger variability only at higher densities. CBCTp calibrations agreed well with vendor references. DVH‐based dosimetric metrics showed differences generally within 1% for both CBCTp and CBCT when compared with CT. Across institutions, gamma analysis of both CBCTp and CBCT yielded high passing rates (≥ 98.5% at 3%/2 mm). End‐to‐end testing with film dosimetry showed that CBCT‐based plans agreed with measured doses within ± 4%, while CT‐based plans were within ± 3%. Ion chamber measurements showed all dose differences within ± 2.3%, with both CBCTp and CBCT within ± 1.0% of CT.

**Conclusions:**

HyperSight CBCT provides accurate dose calculations when properly calibrated. Phantom‐based validation demonstrated sub‐2% deviations and strong agreement with CT, supporting its clinical use in adaptive radiotherapy.

## 1 INTRODUCTION

Radiotherapy (RT) typically involves patient immobilization, planning CT acquisition, target and organ‐at‐risk (OAR) delineation, treatment planning, and image‐guided delivery. Accurate Hounsfield unit (HU) information from CT is essential for dose calculation. Variations in imaging parameters across institutions may influence Cone‐beam computed tomography (CBCT) image quality and dosimetric accuracy; however, cross‐institutional treatment consistency is generally maintained through appropriate system‐specific calibration. These variations may nevertheless become increasingly relevant in CBCT‐based adaptive radiotherapy (ART) workflows, where consistent image quality and HU stability are critical for reliable dose recalculation.^1^

Image‐guided radiation therapy (IGRT) is a crucial component of modern RT, enhancing treatment precision using improved targeting accuracy and treatment adaptation in response to inter‐fraction anatomical changes. CBCT systems have been routinely employed for patient setup verification, but their application has traditionally been limited by suboptimal image quality and unreliable HU values for dose calculation.^2^

The HyperSight imaging system supports two acquisition modes, CBCT for Planning (CBCTp), optimized for HU fidelity and dose‐relevant image quality, and a daily IGRT mode configured for routine patient setup and alignment. Building on these capabilities, the HyperSight imaging system, a recent advancement in CBCT technology, markedly enhances soft‐tissue contrast, spatial resolution, and HU stability through iterative reconstruction and improved scatter‐correction algorithms.^3^ These improvements raise the possibility of directly using CBCT imaging for adaptive dose calculation, potentially streamlining workflows and reducing reliance on repeated planning CT scans.^4^, ^5^ Moreover, if offline ART were applied to approximately 10% of all treatment fractions delivered annually, the additional yearly cost of the advanced CBCT system could be offset.^6^

Nevertheless, implementing CBCT‐based plan calculation in clinical practice requires thorough verification of its dosimetric accuracy and reliability, particularly when used for adaptive planning or during treatment course dose assessment. Although several studies have investigated CBCT dose fidelity using anthropomorphic phantoms or patient data,^7^, ^8^ only a few have focused on Halcyon with HyperSight. The Halcyon platform is characterized by its fast delivery, closed architecture, and exclusive reliance on CBCT for localization. However, dose calculation and measurement consistency across various institutions have not been evaluated to date.

To bridge this knowledge gap, the present study aimed to validate the dosimetric accuracy of HyperSight CBCT on the Halcyon platform for treatment planning across multiple clinical institutions. We hypothesized that properly calibrated HyperSight CBCT can provide dose calculation accuracy comparable to planning CT, as supported by phantom‐based measurements.

## 2 METHODS

### 2.1 Institutions

This study included three institutions equipped with the HyperSight imaging system on the Halcyon platform: Institution A (Kaohsiung Veterans General Hospital), Institution B (Kaohsiung Show Chwan Memorial Hospital), and Institution C (Tungs’ Taichung MetroHarbor Hospital).

### 2.2 Image acquisition

For both CBCTp and CBCT, the acquisition and reconstruction settings were derived from the system's default protocols and adapted as study‐specific variants rather than being configured from scratch. All CBCTp and CBCT acquisitions were performed at 125 kV with a field of view (FOV) of 538 mm, an in‐plane resolution of 1.05 × 1.05 mm^2^, and a tube current‐time product of approximately 800 mAs. CBCT images were acquired using a vendor‐provided pelvic‐mode with 409 projections and a nominal slice thickness of 2.0 mm, whereas CBCTp images were acquired using a vendor‐provided head‐mode with 701 projections and a nominal slice thickness of 3.0 mm. Image reconstruction for both CBCTp and CBCT was performed using the same iCBCT Acuros reconstruction framework. CT scanners at each institution with their routine clinical imaging parameters, as summarized in the supplementary Table S1.

### 2.3 HU‐to‐density calibration curve comparison

HU‐to‐density curves were obtained from the three institutions (referred to as Institutions A, B, and C henceforth) to investigate cross‐institutional calibration curve variation. Each institution used 2 imaging systems: an institution‐specific CT scanner and a Varian Halcyon linear accelerator equipped with HyperSight CBCT imager (Varian Medical Systems, Palo Alto, CA, USA). The institution‐specific configurations are presented in Table 1.

**TABLE 1:** Institution‐specific CT scanner and calibration phantom models.

Institution ID	CT Scanner	Phantom model
A	GE Discovery CT590 RT	Gammex 467 Tissue Characterization Phantom ^#^
B	Philips Brilliance Big Bore	Advanced Electron Density Phantom ^##^
C	Siemens Somatom Definition AS	Model 062 M Electron Density Phantom ^##^

Abbreviations: CT, computed tomography; GE, General Electric; ID, identity; RT, radiotherapy.

^#^ Sun Nuclear Corporation Melbourne, FL, USA.

^##^ Computerized Imaging Reference Systems, Sun Nuclear Corporation, Norfolk, VA, USA.

Each phantom contained inserts designed to simulate densities of various tissues (e.g., lung, adipose, muscle, and bone). Calibration scans were performed on CT scanners of each institution using routine clinical imaging parameters (typically 120 kVp). In contrast, all HyperSight acquisitions were performed using a fixed tube potential of 125 kVp and reconstructed using the iCBCT Acuros. iCBCT Acuros is designed to improve image quality and HU consistency for dose calculation purposes.

The same CIRS (Computerized Imaging Reference Systems, Sun Nuclear Corporation, Norfolk, VA, USA) Model 062 M Phantom was scanned on all three Halcyon platforms equipped with HyperSight CBCT under harmonized acquisition protocols to facilitate cross‐institutional HyperSight comparison. To approximate full scatter conditions during CBCT HU evaluation, the CIRS Model 062 M Phantom was positioned between 5‐cm‐thick water‐equivalent slabs placed anteriorly and posteriorly for all CBCT image acquisitions. HU were extracted from axial images using standardized circular regions of interest (ROIs) placed within each phantom insert. To minimize partial volume effects and ensure reproducibility, the ROI diameter was set to approximately 70% of the physical insert diameter, centered within the insert on the axial slice that passed through its central axis. The extracted HU values were then mapped to the reference mass density values provided by the phantom manufacturer. These values were then compared to assess scanner consistency and alignment with manufacturer reference curves. As shown in Figure 3(b), the measured HU values were comparable to those reported in the manufacturer‐provided IEC type test documentation for the Halcyon and Ethos systems, which were obtained using CBCT‐specific electron density phantoms.^9^

### 2.4 Treatment planning and dosimetric evaluation

CT images of density calibration phantoms from each institution were used to simulate various tissue‐equivalent conditions for dosimetric comparisons. Specifically, representative low‐density (LN300, Lung Inhale), medium‐density (Solid Water, Breast 50/50), and high‐density (Cortical Bone, Dense Bone 1250) materials within the phantoms were contoured and used as target structures.

In addition, STEEV end‐to‐end verification phantom (CIRS Inc., Norfolk, VA, USA) was scanned to evaluate dose calculation performance in a more anatomically realistic scenario. Within the STEEV phantom, 2 target structures were delineated: one located centrally to simulate deep brain tissue, and another positioned over the cranial bone to assess performance in high‐density regions.

All treatment plans used volumetric modulated arc therapy (VMAT) with two arcs for brain and three arcs for cranial bone regions. All plans were initially generated and optimized on the respective planning CT, which served as the reference standard. The Eclipse™ AcurosXB algorithm was used for dose calculation using a 6‐MV flattening‐filter‐free (FFF) photon beam. Each imaging modality used its respective site‐specific HU‐to‐density calibration curve.

The original treatment plan based on CT scans was directly transferred to CBCTp or CBCT images for dose recalculation without any reoptimization or modification to the plan parameters. All ROI contours delineated on the CT images were then rigidly registered and propagated to the CBCTp or CBCT images to preserve structural alignment. Finally, DVH‐based dosimetric metrics, including mean dose (D_mean_), D_95_, and D_2_, were recorded for each target in the density phantoms, whereas two‐dimensional (2D) and three‐dimensional (3D) gamma analyses were performed for the STEEV phantom to assess spatial dose agreement across image sets.

The evaluated dose distributions were analyzed using Verisoft version 7.2 (PTW, PTW‐Freiburg, Freiburg, Germany). The local gamma analyses were performed using both 2D and 3D approaches under multiple criteria, including 3% dose difference/3 mm distance‐to‐agreement, 3%/2 mm, and 2%/2 mm, with a passing threshold of 95%.^10^ In addition, a stricter 2%/1 mm criterion was applied with a 90% passing threshold. A low‐dose threshold of 10% relative to the maximum dose in the reference distribution was used to exclude low‐dose regions from the analysis. All gamma passing rates were recorded for subsequent comparison.

### 2.5 End‐to‐End dose validation

A STEEV phantom and an Alderson RANDO® anthropomorphic phantom (The Phantom Laboratory, Salem, NY, USA) were used to simulate clinically relevant human tissue geometry and heterogeneity to experimentally validate dose calculation accuracy on various imaging modalities. Using an anthropomorphic head and thorax phantom embedded with EBT3 Gafchromic film (Ashland Inc., NJ, USA) and an ion chamber as dosimeters, we performed phantom‐based dose verification to assess the agreement between CT/CBCT‐based dose computation and actual measured values. In this study, EBT3 film was intentionally used for point dose verification at predefined anatomical locations rather than for a full 2D planar dose evaluation. Although EBT3 film is commonly employed for planar dose measurements, it has also been widely used for absolute point dose verification and localized dosimetry in RT, particularly in surface, buildup, and in‐vivo dose measurements.^11^, ^12^ The present dosimetric validation focused on absolute dose accuracy at clinically relevant locations, while avoiding additional uncertainties associated with full planar film analysis, such as scanner nonuniformity, orientation dependence, and extensive calibration requirements.^13^ The STEEV phantom focuses on the head region and allows integration of dosimetric inserts, including EBT3 film and an ionization chamber (PTW 31010 semiflex), enabling high‐precision measurements in cranial structures. The RANDO phantom represents an entire human torso with anatomically segmented structures. It was primarily used for dose measurements in the heart and lungs.

Three treatment plans, each generated using a distinct imaging modality (CT, CBCTp, and CBCT), were created for the phantom. These plans were developed using the same planning optimization parameters and beam geometry. Each imaging modality used its respective site‐specific HU‐to‐density calibration curve. The plans were calculated using the Eclipse™ Acuros algorithm with a 6‐MV FFF photon beam. Dose delivery was performed using a Halcyon linear accelerator.

**FIGURE 1:**
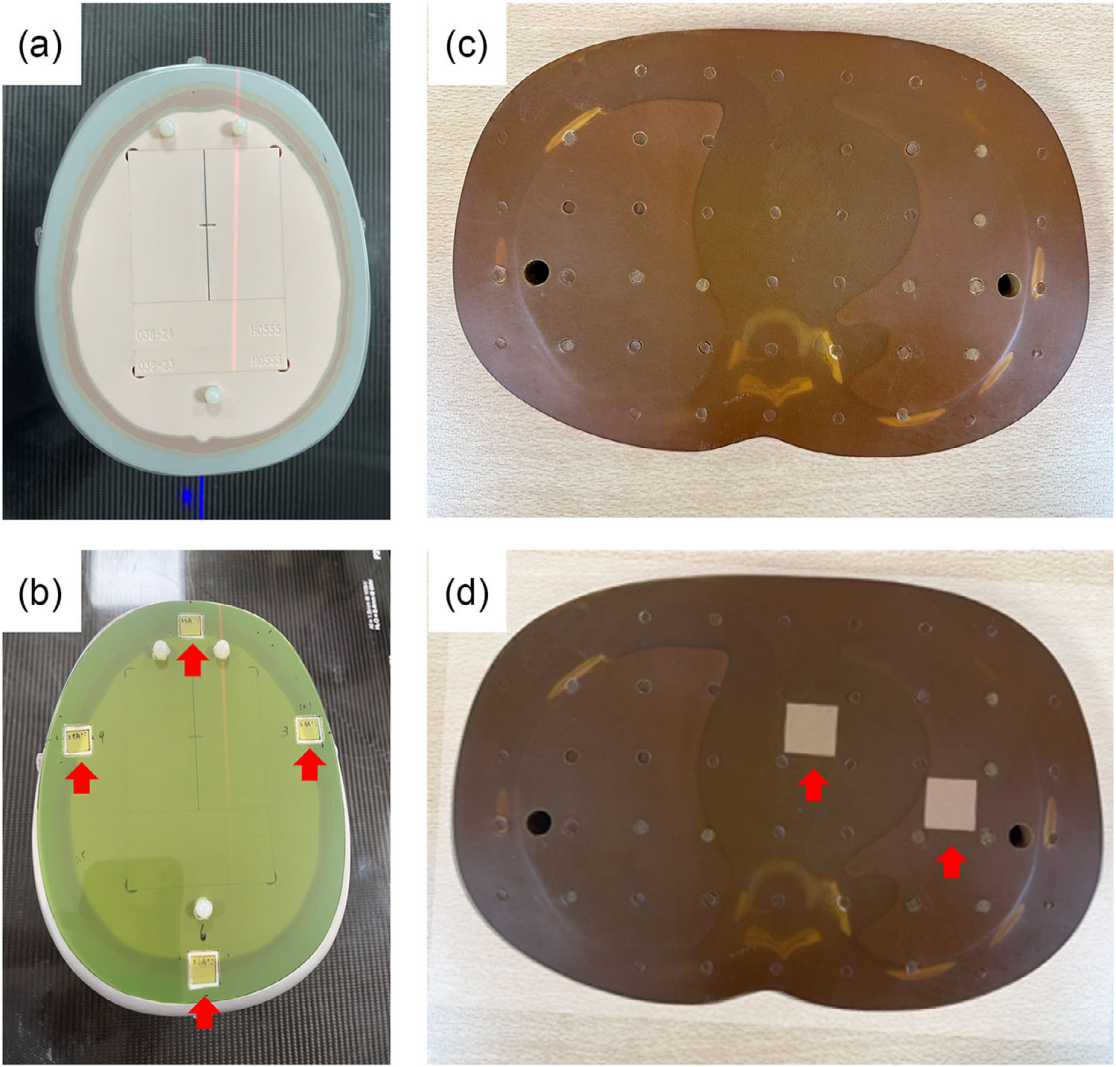
STEEV and RANDO phantoms used for radiation dose verification. (a) Internal view of the STEEV phantom, demonstrating the cavity structure designed to accommodate modular inserts for dosimetric and geometric verification. (b) EBT3 film overlays were placed at four distinct positions on the STEEV phantom, with their locations indicated by red arrows for point dose measurement. The final reported value was calculated as the average of the four measurement points. (c) Transverse cross‐sectional view of the thorax region in the RANDO phantom, demonstrating the internal structure. (d) Placement of EBT3 films at 2 designated positions within the phantom (red arrows), configured for dose measurement and verification.

**FIGURE 2:**
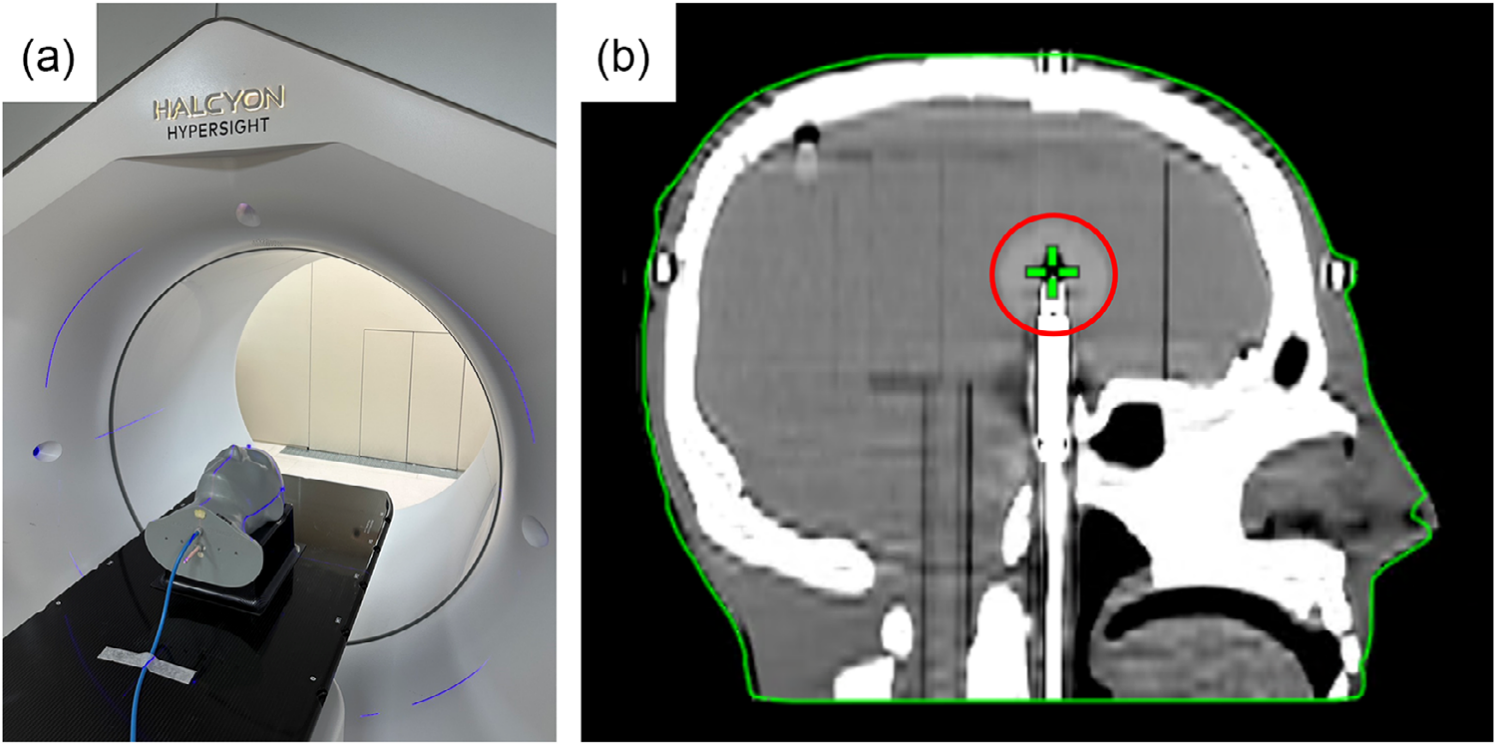
Dose measurement setup using the STEEV phantom on a Halcyon platform. (a) STEEV anthropomorphic phantom positioned on the Halcyon linear accelerator couch for kV imaging in the HyperSight system. (b) Corresponding sagittal reconstructed image demonstrating the ion chamber insert aligned with the isocenter, used for point dose measurement and dosimetric verification, with the red circle indicating the measurement location.

Dosimetric validation was performed at 4 anatomical locations, as illustrated in Figures 1 and 2:

1. Brain region:

A PTW 31010 semiflex small‐volume ionization chamber was positioned at the geometric center for point dose verification. Before dose measurement, CBCT imaging was used to correct setup errors. The chamber was inserted into a dedicated phantom channel and cross‐calibrated against reference measurements from a traceable Accredited Dosimetry Calibration Laboratory (ADCL).

2 Cranial bone region (peripheral high‐density structure):

A 1 × 1 cm^2^ EBT3 film was placed in the axial plane, positioned within the cortical bone region to evaluate dose accuracy in high‐density areas.

3 Heart region (central soft tissue surrounded by heterogeneous thoracic structures) and lung region (low‐density parenchyma with high‐density rib boundaries).

A 2 × 2 cm^2^ film was placed in the axial plane.

For film dosimetry, EBT3 films were positioned in the axial plane of interest within both the STEEV and RANDO phantoms. Following irradiation, the films were scanned 24 hours later using an EPSON 10000XL flatbed scanner (Seiko Epson Corp., Nagano, Japan). Dose calibration was performed with a separate set of films irradiated to known dose levels (result in Figure 4). The procedures for film scanning and calibration were carried out according to previously described methodology.^14^ Ion chamber measurements were conducted at the center of the target region.

The measured doses using film and ion chamber were then compared with corresponding calculated doses from CT, CBCTp, and CBCT plans. The absolute dose and percent dose difference were calculated to evaluate the dosimetric accuracy of the CBCT‐based planning workflow relative to the CT reference.

## 3 RESULTS

### 3.1 Calibration curve comparison

**FIGURE 3:**
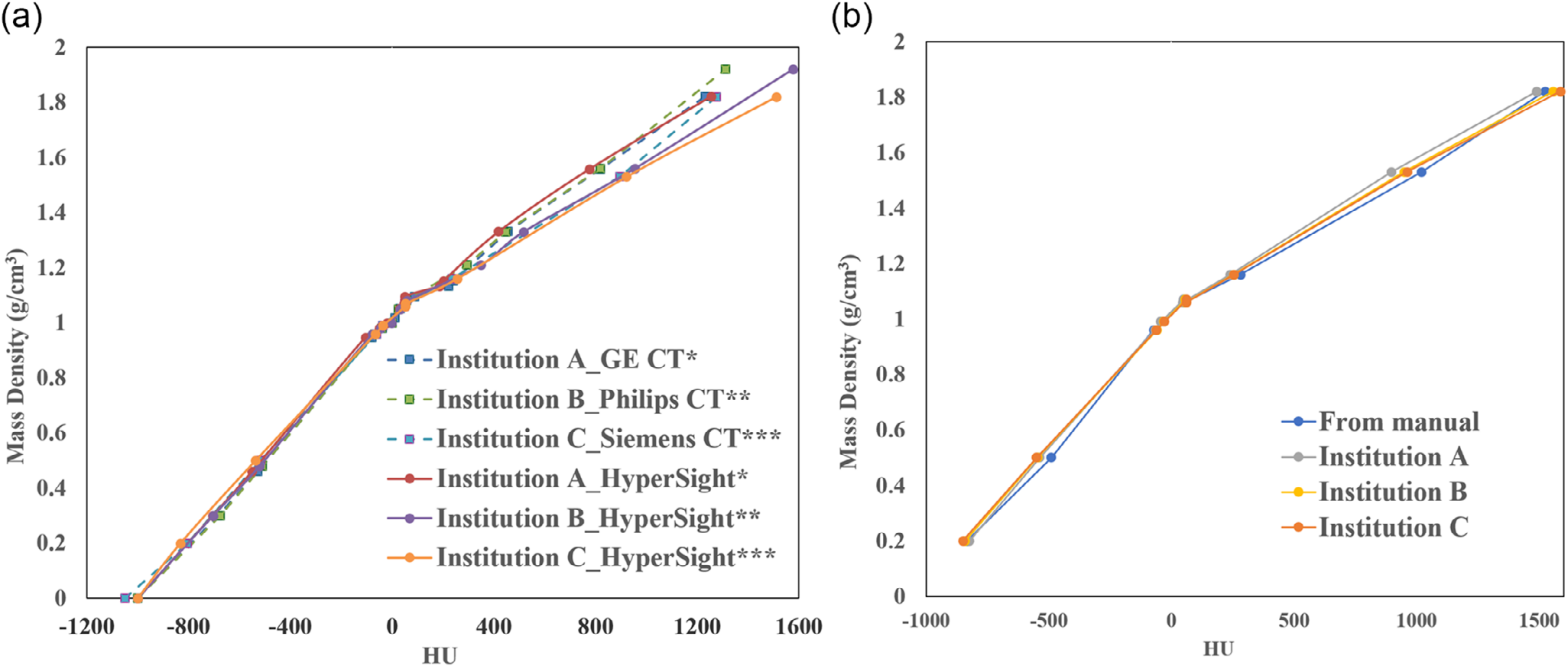
(a) HU‐to‐mass density calibration curves for CT and HyperSight CBCT systems. (b) HU‐to‐mass density calibration curves derived from HyperSight CBCT images of the CIRS Model 062 M Phantom. Data were obtained across three clinical institutions. ^*^Gammex 467 Tissue Characterization Phantom, ^**^Advanced Electron Density Phantom, and ^***^ Model 062 M Electron Density Phantom. Abbreviations: CBCT, Cone‐beam computed tomography; CT, computed tomography; HU, Hounsfield unit.

Figure 3(a) illustrates the HU‐to‐mass density calibration curves obtained from 3 independent clinical institutions (A, B, and C), each using a separate CT scanner, with tube voltages (120 or 125 kVp), and calibration phantoms. The CT and HyperSight CBCT calibration curves were generated for each institution. The CT‐based calibration data were represented by discrete square markers connected by dashed lines, whereas HyperSight CBCT data were plotted as continuous lines.

The HU‐to‐density relationship preserved strong linearity across all datasets. However, noticeable inter‐institutional variability was observed, particularly in the higher HU regions (> 1.4 g/cm^3^). Despite these variations, HyperSight CBCT calibration curves across institutions exhibited acceptable consistency with their respective CT curves in low‐ to mid‐density regions, supporting the robustness of the iterative reconstruction algorithm employed by HyperSight. The observed deviations in high‐density regions emphasize the crucial role of site‐specific calibration if high‐Z implants or dense bony structures are involved in dose‐critical regions.

Figure 3(b) presents the HU‐to‐mass density relationship for HyperSight CBCTp images across the 3 institutions using the same phantom model (062 M). All curves, including the vendor‐provided reference, demonstrated strong alignment.^9^ This suggests that, under standardized phantom conditions, HyperSight CBCT calibration is reproducible across various institutions.

### 3.2 Treatment planning and dosimetric evaluation

Table 2 summarizes the DVH‐based dosimetric metrics D_mean_, D_95_, and D_2_ for various density inserts, comparing dose calculations based on CT, CBCTp, and CBCT across three institutions. Across all institutions and heterogeneous density conditions, dose calculations based on CBCTp and CBCT demonstrated close agreement with CT, with deviations within approximately 1% for all evaluated metrics. ​

**TABLE 2.** Comparison of ROI dose metrics for various density inserts across three institutions (A–C). Mean dose (D_mean_), D_95_, and D_2_ are reported in cGy; percentage differences are calculated relative to CT.

Institution	ROI	Mass density, g/cm^3^	D_mean_					D_95_					D_2_				
			CT	CBCTp	CBCT	ΔCBCTp (%)	ΔCBCT (%)	CT	CBCTp	CBCT	ΔCBCTp (%)	ΔCBCT (%)	CT	CBCTp	CBCT	ΔCBCTp (%)	ΔCBCT (%)
A	LN 300	0.3	99.5	99.5	99.0	0.0	−0.5	97.8	97.5	98.7	−0.3	0.9	100.9	100.8	101.3	−0.1	0.4
	Solid Water	1.017	97.8	97.9	97.7	0.1	−0.1	96.6	96.5	96.5	−0.1	−0.1	99.2	99.7	99.6	0.5	0.4
	Cortical Bone	1.823	95.5	95.8	95.6	0.3	0.1	93.7	93.9	93.8	0.2	0.1	99.2	99.6	99.7	0.4	0.5
B	LN 300	0.3	101.4	101.6	101.9	0.2	0.5	98.2	98.2	99.4	0	1.2	103.4	103.8	103.9	0.4	0.5
	Solid Water	1.021	101.0	101.3	101.5	0.3	0.1	98.6	98.8	99.1	0.2	0.5	102.9	103.4	104.1	0.5	1.2
	Cortical Bone	1.924	101.0	101.4	101.6	0.4	0.6	99.1	99.2	99.8	0.1	0.7	102.9	103.4	103.6	0.5	0.7
C	Lung Inhale	0.2	100.8	100.2	100.1	−0.6	−0.7	97.1	96.2	97.5	−0.9	0.4	102.8	102.3	102.5	−0.5	−0.3
	Breast 50/50	0.99	100.3	99.9	99.7	−0.4	−0.6	97.9	97.4	97.2	−0.5	−0.7	102.3	102.2	102.3	−0.1	0.0
	Dense Bone 1250	1.82	100.4	100.2	100.0	−0.2	−0.4	98.4	97.8	98.0	−0.6	−0.4	102.1	101.9	102.5	−0.2	0.4

Abbreviations: CBCT, Cone‐beam computed tomography; CBCTp, CBCT for planning; CT, computed tomography; ROI, region of interest.

2D and 3D dose comparisons on the STEEV phantom were performed for brain and cranial bone target locations. Table 3 summarizes the gamma passing rates obtained across the three institutions. All dose calculations were based on using CT, CBCTp, and CBCT images. Across all the three institutions, both CBCTp and CBCT achieved high agreement with CT‐based reference plans under standard criteria (≥ 98.5% for 3%/3 mm and 3%/2 mm) in both 2D and 3D gamma analyses. Under more stringent evaluation criteria (2%/2 mm and 2%/1 mm), dose calculations based on CBCTp and CBCT demonstrated generally high 3D gamma passing rates across anatomical regions, with most values exceeding 95%.​ Overall, the results showed comparable performance between CBCTp and CBCT under these strict criteria.

**TABLE 3:** Two‐dimensional (2D) and three‐dimensional (3D) gamma passing rates (%) for brain and cranial bone targets in the STEEV phantom across the three institutions under multiple gamma criteria.

Institution	Location	Modality	3%/3 mm		3%/2 mm		2%/2 mm		2%/1 mm	
			2D	3D	2D	3D	2D	3D	2D	3D
A	Brain	CBCTp	100.0	100.0	99.5	99.9	99.5	99.9	95.2	98.0
		CBCT	100.0	100.0	99.8	100.0	99.8	100.0	97.3	99.0
	Cranial bone	CBCTp	100.0	99.7	99.2	98.9	99	97.4	95.6	92.8
		CBCT	100.0	99.7	99.5	99.4	97.3	98.0	94.4	95.1
B	Brain	CBCTp	100.0	100.0	98.9	99.9	98.7	99.8	94.2	97.7
		CBCT	100.0	100.0	99.7	99.9	99.6	99.7	96.1	98.4
	Cranial bone	CBCTp	100.0	99.5	98.9	98.8	98.4	97.9	94.4	95.4
		CBCT	100.0	99.9	99.4	99.3	97.4	98.9	91.3	96.6
C	Brain	CBCTp	100.0	100.0	99.8	99.5	99.8	99.5	97.7	98.4
		CBCT	100.0	100.0	99.5	99.3	99.4	99.3	95.0	96.4
	Cranial bone	CBCTp	100.0	99.5	99.8	99.1	99.8	96.6	97.9	91.2
		CBCT	100.0	100.0	100	99.1	99.6	97.2	97.3	93.6

Abbreviations: CBCT, Cone‐beam computed tomography; CBCTp, CBCT for planning; CT, computed tomography.

Overall, the 3D gamma results were consistent with the corresponding 2D gamma findings, indicating good volumetric dose agreement throughout the irradiated volume. These results suggest that both CBCTp and CBCT are suitable for dose calculation in this phantom‐based evaluation. The lowest observed gamma passing rate (91.2%) occurred at the cranial bone location in Institution C under the 2%/1 mm criterion, which may reflect increased sensitivity to setup or image registration variations in high‐density regions under strict gamma thresholds.

### 3.3 End‐to‐End dose validation

**FIGURE 4:**
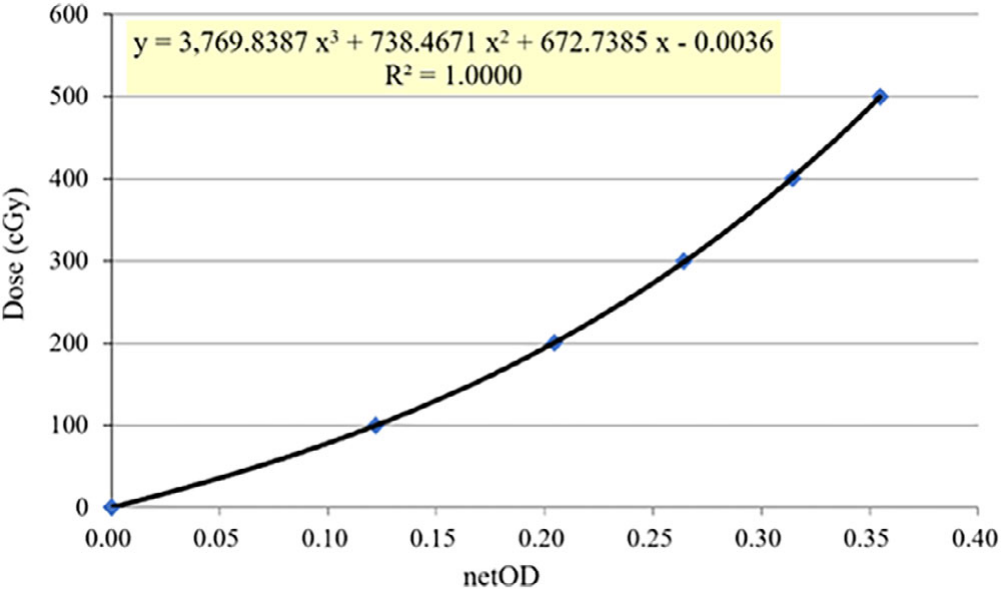
The EBT3 film sensitometric curve.

Figure 4 illustrates the sensitometric curve of the EBT3 film, which was fitted with a third‐order polynomial. Reported parameters included the fit coefficients and the coefficient of determination (R^2^). The observed R^2^ values remained within 0.9998–1.0000, confirming the high stability of the EBT3 film measurements.

The dosimetric validation was conducted using ion chamber and film measurements at 2 anatomical locations across all three institutions. As demonstrated in Table 4, film dosimetry on anthropomorphic phantoms was used to perform point dose comparisons at the cranial bone, heart, and lung across the three institutions. The dose calculations were performed using CT, CBCTp, and CBCT images. CT‐based plans revealed close agreement with measurements (within approximately 3%). CBCTp and CBCT also demonstrated acceptable accuracy, with most differences within ± 4%. The lung region demonstrated the largest variations, which may be attributed to greater heterogeneity. Across all sites, CBCTp and CBCT dose calculations were within ± 2% of CT, supporting its feasibility for clinical use with proper calibration.

**TABLE 4:** Comparison of planned and measured doses using EBT3 film at cranial bone, heart, and lung locations across the 3 institutions and imaging modalities.

Institution	Modality	Location	Planned dose (cGy)	Measured dose (cGy)	Planned/Measured Diff (%)	Diff from CT (%)
A	CT	Cranial bone	303.4	308.0	1.5	NA
		Heart	303.8	311.5	2.5	NA
		Lung	302.7	308.9	2.1	NA
	CBCTp	Cranial bone	303.6	304.2	0.2	−1.3
		Heart	303.2	315.0	3.9	1.4
		Lung	303.1	314.8	3.9	1.8
	CBCT	Cranial bone	303.8	304.5	0.2	−1.3
		Heart	303.1	309.3	2.1	−0.5
		Lung	303.8	315.0	3.7	1.6
B	CT	Cranial bone	302.9	307.7	1.6	NA
		Heart	306.3	315.1	2.9	NA
		Lung	304.7	312.1	2.4	NA
	CBCTp	Cranial bone	303.0	304.9	0.6	−1.0
		Heart	306.3	313.2	2.3	−0.6
		Lung	304.7	312.0	2.4	0.0
	CBCT	Cranial bone	301.0	305.2	1.4	−0.2
		Heart	302.2	308.3	2.0	−0.8
		Lung	304.5	313.6	3.0	0.6
C	CT	Cranial bone	305.0	305.4	0.1	NA
		Heart	306.3	303.3	−1.0	NA
		Lung	304.7	297.9	−2.3	NA
	CBCTp	Cranial bone	307.7	309.0	0.4	0.3
		Heart	306.3	307.2	0.3	1.3
		Lung	304.7	297.1	−2.5	−0.2
	CBCT	Cranial bone	308.5	306.0	−0.8	−0.9
		Heart	302.2	296.9	−1.7	−0.8
		Lung	304.5	294.0	−3.5	−1.2

Abbreviations: CBCT, Cone‐beam computed tomography; CBCTp, CBCT for planning; CT, computed tomography; NA, not applicable.

The ion chamber measurements were conducted across the three institutions using CT, CBCTp, and CBCT imaging. As presented in Table 5, the dose differences between planned and measured values ranged from −0.3% to 2.3%, with all deviations within an acceptable range. CT plans generally demonstrated acceptable agreement with measured doses (0.7% to 1.9%). CBCTp and CBCT plans demonstrated comparable accuracy, with dose differences relative to CT within ± 1.0%.

**TABLE 5:** Comparison of planned and measured doses using an ion chamber at the brain location across the three institutions and imaging modalities.

Institution	Modality	Planned dose (cGy)	Measured dose (cGy)	Planned/Measured Diff (%)	Diff from CT (%)
A	CT	302.3	308.1	1.9	NA
	CBCTp	301.3	308.3	2.3	0.4
	CBCT	301.9	306.9	1.6	−0.3
B	CT	299.6	303.8	1.4	NA
	CBCTp	300.4	304.4	1.3	−0.1
	CBCT	301.8	305.1	1.1	−0.3
C	CT	300.5	302.6	0.7	NA
	CBCTp	302.1	301.7	−0.1	−0.8
	CBCT	304.1	303.2	−0.3	−1.0

Abbreviations: CBCT, Cone‐beam computed tomography; CBCTp, CBCT for planning; CT, computed tomography; NA, not applicable.

## 4 DISCUSSION

The end‐to‐end measurement results of this study demonstrated that a properly calibrated and configured Halcyon with HyperSight CBCT system, within the treatment planning system, can achieve high dosimetric accuracy for plan calculation. The ion chamber measurements in STEEV phantoms remained within an overall maximum deviation of ± 3% across all institutions, consistent with the clinical tolerance recommended by the American Association of Physicists in Medicine (AAPM) Medical Physics Practice Guideline.^15^ After applying appropriate density calibration in Eclipse, the dose calculations based on the three imaging modalities demonstrated differences of less than 1% for DVH‐based dosimetric metrics for all ROIs, all plans met TG‐218 QA criteria with > 95% gamma passing (3%/2 mm).^10^ These findings highlight the stability of dose delivery in CBCT‐based ART workflows, supporting their clinical reliability.

Several recent studies have evaluated the dosimetric performance of HyperSight CBCT on Halcyon platforms, primarily focusing on the feasibility of dose calculation using CBCT images and ART workflows. Phantom‐based investigations have demonstrated that HyperSight CBCT improves HU fidelity and dose calculation accuracy compared with earlier Halcyon imaging systems, with gamma passing rates typically exceeding 95–98% across multiple anatomical sites.^7^ At the institutional level, Nelson et al. performed a comprehensive phantom benchmark and end‐to‐end delivery verification, showing that dose calculations based on HyperSight CBCT images agreed with CT simulation within approximately 1%, supporting the feasibility of CBCT‐only treatment planning.^16^ Similarly, MacDonald et al. demonstrated the feasibility of CT simulation‐free ART workflows using HyperSight CBCT within an Ethos‐based platform, emphasizing workflow efficiency and clinical implementation.^8^ Other studies have further confirmed acceptable HU accuracy and dosimetric agreement for CBCT‐only ART using HyperSight, including longitudinal HU stability assessments.^17^

While these studies collectively establish the feasibility and accuracy of HyperSight CBCT for dose calculation, they predominantly rely on single‐institution data, vendor‐oriented phantom evaluations, or a single CBCT acquisition mode. In contrast, the present study extends the existing literature in several important aspects. First, it provides a multi‐institutional evaluation involving three independent clinical centers, explicitly addressing inter‐institutional variability in HU‐to‐density calibration and its dosimetric impact. Second, unlike previous studies that focused primarily on CBCT, this work directly compares CBCTp and CBCT acquisition mode within the same reconstruction framework. Finally, our end‐to‐end validation integrates absolute dose measurements across heterogeneous anatomical regions, enabling a targeted assessment of CBCT‐based dose calculation performance.

Although the present results demonstrate that CBCT can achieve clinically acceptable accuracy for dose calculation, an initial planning image remains indispensable in RT. In current clinical practice, CT image is most used to provide the geometric reference, accurate target delineation basis, and comprehensive localization framework required to safely initiate treatment.^8^ The requirements placed on CBCT image quality depend strongly on its intended use. When CBCT is used primarily for pre‐treatment setup verification, the demands on image quality, HU accuracy, and reconstruction fidelity are relatively modest. In contrast, CBCT‐based dose calculation requires more stringent acquisition and reconstruction conditions, including appropriate kVp selection, sufficient effective mAs, and reconstruction algorithms optimized for HU stability and scatter correction.^4^, ^18^ CBCTp was developed to support these planning‐related requirements by enabling planning‐oriented image acquisition and reconstruction within the treatment environment. When acquisition and reconstruction parameters are closely matched, CBCTp and CBCT demonstrate comparable dose calculation accuracy; however, CBCT workflows typically lack planning‐specific capabilities such as flexible selection of planning‐oriented slice thickness, redefinition of the treatment isocenter, and laser‐based patient localization.^19^ By providing a structured, planning‐oriented framework, CBCTp supports treatment planning and replanning in adaptive scenarios where such workflow flexibility is required, and should be viewed as a practical extension of CBCT‐based adaptive workflows for selected clinical applications.

Furthermore, the results highlight that the improved HU stability of HyperSight CBCT imaging was crucial in reducing density assignment errors, which has been a major limitation in CBCT dose calculation.^19^, ^20^ Compared with legacy CBCT systems, where image artifacts and scatter distort soft tissue contrast, the enhanced reconstruction algorithms of the HyperSight system helped preserve anatomical accuracy.^19^, ^21^

The HU values in CT are influenced by several factors, including tube voltage (kVp), reconstruction algorithms, scanner type, and phantom design.^22^ The HU‐to‐density curve in this study was collected from 4 distinct vendors through three medical institutions. Tube voltages applied were 120 or 125 kVp, and each site employed a separate calibration phantom. These variations led to observable differences in HU values corresponding to the same physical density. Although the overall relationship between density and HU remained linear, noticeable deviations were observed in the high‐density range, consistent with previous studies.^18^ Notably, the HU‐to‐mass density curves obtained with the HyperSight CBCT system showed strong consistency across the three institutions. Such inter‐institutional concordance, consistent with previous reports, further supports the reproducibility of density calibration.^1^ This consistency suggests that treatment plans can be reliably created and adapted using images from various institutions, supporting the feasibility of direct patient transfer between centers.

The findings were consistent with prior studies indicating that variations between HU‐to‐density calibration curves exert negligible influence on the resulting dose distributions.^23^ In this study, calibration curves from three institutions were compared, and dose calculations were performed based on both CT and CBCT‐based images across targets of varying densities. The analysis revealed that differences in DVH‐based dosimetric metrics for the ROI among the three calibration curves were all less than 1.0%. Importantly, these differences were well below levels that would be expected to influence adaptive replanning decisions in routine clinical practice, indicating that dose calculations derived from CT, CBCTp, and CBCT images would be expected to result in the same adaptive decision under the conditions studied. Furthermore, 2D and 3D gamma analyses with a passing criterion of 2%/2 mm demonstrated that all treatment plans achieved a passing rate greater than 95%, with most cases exceeding 98%. The inclusion of 3D gamma analysis enables voxel‐wise assessment of dose agreement, increasing sensitivity to localized discrepancies in steep‐gradient and heterogeneous regions. Although our results demonstrated that HyperSight CBCT maintained high dose reproducibility across varying HU‐to‐density calibration curve settings, we still recommend that each institution should establish and periodically verify its own calibration curve to comply with quality assurance guidelines and facilitate long‐term performance monitoring. This approach not only meets regulatory requirements but also serves as a baseline for identifying potential shifts in system behavior over time, highlighting the robustness and stability of current clinical workflows.

Maximum differences between CT‐ and CBCT‐based dose verifications were within 1% for ion chamber measurements and 2% for EBT3 film measurements, indicating acceptable agreement. Regardless of the imaging modality, the absolute dose deviations measured by the ion chamber generally ranged from 1.5% to 2%, which was consistent with previously published studies.^8^ For film dosimetry, most measurements demonstrated dose differences within 3%. This discrepancy in film dosimetry results may be attributed to setup‐related uncertainties or inherent limitations in film dosimetry, such as air gaps between the film and the phantom surface. These results confirm the feasibility of using CBCT‐based images for both image guidance and primary dose calculation.

One of the key findings lay in the excellent agreement between CBCT‐calculated doses and phantom‐embedded detector measurements, serving as a simulation of in vivo dose verification. This was particularly remarkable considering the anatomical complexity and density heterogeneity present in the thoracic and cranial regions. The results demonstrated that HyperSight CBCT systems were capable of not only providing accurate anatomical visualization but also supporting accurate dose calculation.

The high dosimetric accuracy demonstrated by HyperSight CBCT has notable clinical implications. Most remarkably, it supports the use of CBCT as a single modality for both patient positioning and dose calculation in ART. This integration reduces the reliance on repeat CT simulation, a process that is resource‐intensive and logistically challenging. In resource‐limited settings, using in‐room CBCT for both imaging and treatment planning can streamline workflows, reduce costs, and expand access to ART techniques. Ultimately, this enables instant response and realizes individualized treatment without sacrificing accuracy or safety.

In cases of high tissue heterogeneity or tumor regression,^24^ HyperSight CBCT provides superior image quality with more stable HU values and improved soft‐tissue contrast than synthetic CT, enabling more accurate depiction of daily anatomical changes such as tumor shrinkage, bowel gas variation, and body contour alterations.^18^ With proper HU calibration, HyperSight images are appropriate for direct dose calculation and demonstrate acceptable consistency. By enabling clearer visualization of the organs with more accurate contours, thereby enhancing the precision of dose assessment.^25^

Beyond dose calculation accuracy, ART decisions also depend critically on anatomical delineation and contouring reliability.^26^ Although contouring uncertainty was not explicitly evaluated in this study, the improved image quality and HU stability observed in HyperSight CBCTp and CBCT may, in this context, contribute to clearer soft‐tissue boundaries and more consistent contouring in CBCT‐based adaptive workflows.^27^ While direct quantitative assessment of contouring variability was beyond the scope of this phantom‐based study, prior image quality studies of HyperSight CBCT have demonstrated improved soft‐tissue contrast and reduced artifacts, which are expected to facilitate more reliable anatomical delineation in ART workflows.^5^, ^28^ Future studies incorporating quantitative contouring variability assessments would be valuable to further investigate this aspect.

Another key component of CBCT‐based ART is deformable image registration (DIR) between the planning CT and daily CBCT. Image quality, artifact level, and HU consistency are known to influence DIR performance and subsequent dose accumulation accuracy.^5^ Although DIR was not evaluated in the present work, the demonstrated dosimetric consistency of HyperSight CBCTp and CBCT under controlled conditions suggests potential suitability for DIR‐based adaptive workflows. Further investigations assessing DIR accuracy and dose accumulation using patient datasets are warranted.

However, several limitations of this study must be acknowledged. First, the rigid and static nature of the phantom does not replicate clinically relevant factors such as anatomical deformation, inter‐ and intra‐fraction variability, or patient motion. Second, all imaging acquisitions were performed under standardized and controlled conditions, and clinically relevant sources of variability such as patient size, off‐center positioning, metal implants, lower mAs, and increased scatter were not explicitly evaluated. Variations in these factors are known to influence CBCT image quality, HU stability, and resultant dose accuracy.^29^, ^30^, ^31^, ^32^ Although prior image quality studies suggest that HyperSight maintains improved noise and contrast performance at lower effective mAs compared to conventional CBCT systems, systematic dosimetric assessment under such non‐ideal imaging conditions remains warranted.^33^ We agree that extending this evaluation to patient cases or deformable scenarios would further enhance clinical relevance and represent an important direction for future work. Finally, while point dose agreement was excellent, volumetric dose accuracy in clinical practice may be influenced by anatomical changes and the performance of contour propagation and DIR. Although the present results support the feasibility of CBCT‐based adaptive planning under controlled conditions, further studies incorporating patient datasets are needed to assess DIR accuracy, dose accumulation, and their impact on adaptive decision‐making.

## 5 CONCLUSIONS

Dose calculations using HyperSight CBCT on Halcyon differed by < 2% from CT‐based calculations in phantom‐based in vivo simulations. These findings support its clinical use in ART workflows, including mid‐treatment assessment, on‐demand plan adaptation, and dose auditing.

## AUTHOR CONTRIBUTION

**Chih‐Yuan Lin**: conceptualization, data curation, formal analysis, investigation, methodology, validation, visualization, writing—original draft. **Yi‐Ling Chen**: conceptualization, investigation, methodology, project administration, resources, validation, writing—review and editing. **Chia‐Chi Chang**: conceptualization, project administration, resources, validation, writing—review and editing. **Yin‐Hsun Hu**: conceptualization, data curation, investigation, methodology, validation, writing—review and editing. **Chia‐Peng Pan**: conceptualization, methodology, resources, software. **Fang‐Hui Liu**: investigation, resources. **Hsiang‐Ping Chao**: investigation, resources. **Yang‐Wei Hsieh**: investigation, resources, software. **Yu‐Wei Lin, Chi‐Yuan Yeh, Tzu‐Yuan Chao, Shih‐Ming Hsu**: supervision, writing—review and editing.

## FUNDING INFORMATION

The authors received no financial support for this research.

## CONFLICT OF INTEREST STATEMENT

The authors declare that there are no conflicts of interest.

## ETHICS STATEMENT

Ethical approval was not required for this study as no identifiable human subject data were used.

## Supporting information



Supporting Information

## Data Availability

The data supporting the findings of this study are available from the corresponding author upon reasonable request.

## References

[1] Agulles‐Pedros L , MacDonald RL , Cherpak AJ , et al. Multi‐institutional study on image quality for a novel CBCT solution on O‐ring linac. J Appl Clin Med Phys. 2025;26(6):e70023. doi:10.1002/acm2.70023 40048322 PMC12148799

[2] Gong H , Liu B , Zhang G , et al. Evaluation of dose calculation based on cone‐beam CT using different measuring correction methods for head and neck cancer patients. Technol Cancer Res Treat. 2023;22:15330338221148317. doi:10.1177/15330338221148317 36638542 PMC9841465

[3] Wang A , Maslowski A , Messmer P , et al. Acuros CTS: a fast, linear boltzmann transport equation solver for computed tomography scatter—Part II: system modeling, scatter correction, and optimization. Med Phys. 2018;45(5):1914–1925. doi:10.1002/mp.12849 29509973

[4] Sijtsema ND , Penninkhof JJ , van de Schoot AJAJ , et al. Dose calculation accuracy of a new high‐performance ring‐gantry CBCT imaging system for prostate and lung cancer patients. Radiother Oncol. 2025;202:110596. doi:10.1016/j.radonc.2024.110596 39454887

[5] Zhao H , Nelson G , Sarkar V , et al. Comprehensive image quality evaluation and motion phantom studies of an ultra‐fast (6‐second) cone‐beam computed tomography imaging system on a ring gantry linear accelerator. Adv Radiat Oncol. 2025;10(2):101681. doi: 10.1016/j.adro.2024.101681 39717196 PMC11665466

[6] DA Larrotta‐Castillo , Blommestein HM , Kunnen B , et al. Cost‐benefit analysis of advanced CBCT imaging: incremental costs and savings. Radiother Oncol. 2025;211:111106. doi:10.1016/j.radonc.2025.111106 40818490

[7] Wessels C , Strzelecki A , Plamondon M , et al. Technical note: phantom‐based evaluation of CBCT dose calculation accuracy for use in adaptive radiotherapy. Med Phys. 2024;51(10):7492–7499. doi:10.1002/mp.17325 39101716

[8] MacDonald RL , Fallone C , Chytyk‐Praznik K , Robar J , Cherpak A . The feasibility of CT simulation‐free adaptive radiation therapy. J Appl Clin Med Phys. 2024;25(9):e14438. doi:10.1002/acm2.14438 38889325 PMC11492295

[9] Varian Medical Systems I . *Halcyon and Ethos Radiotherapy System IEC Accompanying Documents Type Tests and Procedures*. 2022. https://www.myvarian.com

[10] Miften M , Olch A , Mihailidis D , et al. Tolerance limits and methodologies for IMRT measurement‐based verification QA: recommendations of AAPM Task Group No. 218. Med Phys. 2018;45(4):e53–e83. doi:10.1002/mp.12810 29443390 10.1002/mp.12810

[11] Akbaş U , Kesen ND , Koksal C , Bilge H . Surface and buildup region dose measurements with Markus parallel‐plate ionization chamber, Gafchromic EBT3 film and MOSFET detector for high‐energy photon beams. Advances in High Energy Physics. 2016;2016:8361028.

[12] Dąbrowski R , Drozdyk I , Kukołowicz P . High accuracy dosimetry with small pieces of Gafchromic films. Rep Pract Oncol Radiother. 2018;23(2):114–120. doi:10.1016/j.rpor.2018.01.001 29681774 10.1016/j.rpor.2018.01.001 PMC5908271

[13] Niroomand‐Rad A , Chiu‐Tsao ST , Grams MP , et al. Report of AAPM task group 235 radiochromic film dosimetry: an update to TG‐55. Med Phys. 2020;47(12):5986–6025. doi:10.1002/mp.14497 32990328 10.1002/mp.14497

[14] Lin CY , Shiau AC , Ji JH , et al. A simple method for determining dosimetric leaf gap with cross‐field dose width for rounded leaf‐end multileaf collimator systems. Radiat Oncol. 2018;13(1):222. doi:10.1186/s13014‐018‐1164‐1 30424789 10.1186/s13014-018-1164-1 PMC6234646

[15] Smilowitz JB , Das IJ , Feygelman V , et al. AAPM Medical Physics Practice Guideline 5.a.: commissioning and QA of Treatment Planning Dose Calculations — Megavoltage Photon and Electron Beams. J Appl Clin Med Phys. 2015;16(5):14–34. doi:10.1120/jacmp.v16i5.5768 26699330 10.1120/jacmp.v16i5.5768 PMC5690154

[16] Nelson N , Oare C , Nelson G , Martin T , Huang J , Zhao H . Feasibility of HyperSight CBCT for adaptive radiation therapy: a phantom benchmark study of dose calculation accuracy and delivery verification on the Halcyon. J Appl Clin Med Phys. 2025;26(9):e70245. doi:10.1002/acm2.70245 40926273 10.1002/acm2.70245 PMC12419991

[17] Dusi F , Busato F , Testolin A , et al. Evaluation of the dosimetric accuracy of HyperSight CBCT for CBCT‐only adaptive radiotherapy workflow. Physica Medica. 2025;137:105091. doi:10.1016/j.ejmp.2025.105091 40850155 10.1016/j.ejmp.2025.105091

[18] Duan J , Pogue JA , Stanley DN , et al. Assessing HyperSight iterative CBCT for dose calculation in online adaptive radiotherapy for pelvis and breast patients compared to synthetic CT. J Appl Clin Med Phys. 2025;26(5):e70038. doi:10.1002/acm2.70038 40029696 10.1002/acm2.70038 PMC12059299

[19] Haertter A , Salerno M , Koger B , et al. ACR benchmark testing of a novel high‐speed ring‐gantry linac kV‐CBCT system. J Appl Clin Med Phys. 2024;25(5):e14299. doi:10.1002/acm2.14299 38520072 10.1002/acm2.14299 PMC11087172

[20] Richter A , Hu Q , Steglich D , et al. Investigation of the usability of conebeam CT data sets for dose calculation. Radiat Oncol. 2008;3:42. doi:10.1186/1748‐717X‐3‐42 19087250 10.1186/1748-717X-3-42 PMC2648965

[21] Kunnen B , JAJvdS A , Fremeijer KP , et al. The added value of a new high‐performance ring‐gantry CBCT imaging system for prostate cancer patients. Radiother Oncol. 2024;200:110458. doi:10.1016/j.radonc.2024.110458 39069089 10.1016/j.radonc.2024.110458

[22] Jaafar AM , Elsayed H , Khalil MM , Yaseen MN , Alshewered A , Ammar H . The influence of different kVs and phantoms on computed tomography number to relative electron density calibration curve for radiotherapy dose calculation. Precis Radiat Oncol. 2022;6(4):289–297. doi:10.1002/pro6.1177

[23] Thanh Tai D , Nhu Tuyen P , Duc Tuan H , et al. Scanning protocol influence on relative electron Density‐CT number calibrations and radiotherapy dose calculation for a Halcyon Linac. Radiat. Phys. Chem. 2025;236 : 112760. doi:10.1016/j.radphyschem.2025.112760

[24] Chiou YR , Lin TC , Ji JH , Shiau AC , Huang CH , Liang JA . Large pleural metastases with significant inter‐fractional volume reduction during online adaptive radiotherapy: a case report with dosimetry comparison. Cureus. 2024;16(9):e68407. doi:10.7759/cureus.68407 39360108 10.7759/cureus.68407 PMC11445199

[25] Vaassen F , Hazelaar C , Canters R , Peeters S , Petit S , van Elmpt W . The impact of organ‐at‐risk contour variations on automatically generated treatment plans for NSCLC. Radiother Oncol. 2021;163:136–142. doi:10.1016/j.radonc.2021.08.014 34461185 10.1016/j.radonc.2021.08.014

[26] Brouwer CL , Steenbakkers RJ , Bourhis J , et al. CT‐based delineation of organs at risk in the head and neck region: dAHANCA, EORTC, GORTEC, HKNPCSG, NCIC CTG, NCRI, NRG oncology and TROG consensus guidelines. Radiother Oncol. 2015;117(1):83–90. doi:10.1016/j.radonc.2015.07.041 26277855 10.1016/j.radonc.2015.07.041

[27] Sluijter JH , van de Schoot A , Yaakoubi AE , et al. Evaluation of artificial intelligence‐based autosegmentation for a high‐performance cone‐beam computed tomography imaging system in the pelvic region. Phys Imaging Radiat Oncol. 2025;33:100687. doi:10.1016/j.phro.2024.100687 39802649 10.1016/j.phro.2024.100687 PMC11721864

[28] Robar JL , Cherpak A , MacDonald RL , et al. Novel technology allowing cone beam computed tomography in 6 seconds: a patient study of comparative image quality. Pract Radiat Oncol. 2024;14(3):277–286. doi:10.1016/j.prro.2023.10.014 37939844 10.1016/j.prro.2023.10.014

[29] Mail N , Li F , Lalonde R , Huq MS . The impact of off‐centered positioning on cone beam CT image quality for image‐guided brain SRS treatment. J Appl Clin Med Phys. 2025;26(12):e70388. doi:10.1002/acm2.70388 41290356 10.1002/acm2.70388 PMC12646830

[30] Ordóñez‐Sanz C , Cowen M , Shiravand N , MacDougall ND . CBCT imaging: a simple approach for optimising and evaluating concomitant imaging doses, based on patient‐specific attenuation, during radiotherapy pelvis treatment. Br J Radiol. 2021;94(1124). doi:10.1259/bjr.20210068 10.1259/bjr.20210068 PMC8523193 34282947

[31] Lustermans D , Fonseca GP , Taasti VT , et al. Image quality evaluation of a new high‐performance ring‐gantry cone‐beam computed tomography imager. Phys Med Biol. 2024;69(10) 105018. doi:10.1088/1361‐6560/ad3cb0 10.1088/1361-6560/ad3cb0 38593826

[32] Zhao H , Nelson N , Streitmatter S , et al. Correlation between exposure (mAs) and image quality for a rapid cone‐beam CT on a ring gantry linear accelerator. J Appl Clin Med Phys. 2025;26(12):e70374. doi:10.1002/acm2.70374 41253693 10.1002/acm2.70374 PMC12626742

[33] Kim E , Park YK , Zhao T , et al. Image quality characterization of an ultra‐high‐speed kilovoltage cone‐beam computed tomography imaging system on an O‐ring linear accelerator. J Appl Clin Med Phys. 2024;25(5):e14337. doi:10.1002/acm2.14337 38576183 10.1002/acm2.14337 PMC11087174

