# Supplementary material for: Multi‐institutional validation of hypersight CBCT‐based dose calculation on O‐ring linacs
Source: J Appl Clin Med Phys. 2026 Feb 16;27(2):e70512. doi: 10.1002/acm2.70512 (PMC12909596; doi:10.1002/acm2.70512)
Supplement: Supplementary file 1 — Supporting Information [file ACM2-27-e70512-s001.docx]

Table S1. Acquisition and reconstruction parameters for different CT phantoms across three institutions (A, B, and C).

| Phantom | Institution | kVp | mAs | Reconstruction diameter (mm) | Slice thickness (mm) | In-plane resolution (cm) | Filter type | Kernel |
| --- | --- | --- | --- | --- | --- | --- | --- | --- |
| STEEV | A | 120 | NA | 600 | 1.25 | 0.1172 | Body filter | Standard |
|  | B |  | 450 | 250 | 2 | 0.0488 | UB filter | UB |
|  | C |  | 200 | 500 | 3 | 0.0977 | FLAT filter | D34f |
| RANDO | A |  | 53 | 600 | 2.5 | 0.1172 | Body filter | Standard |
|  | B |  | 300 | 600 | 3 | 0.1172 | B | B |
|  | C |  | 200 | 500 | 3 | 0.0977 | FLAT filter | B31f |
| Gammex 467 | A |  | NA | 600 | 1.25 | 0.1172 | Body filter | Standard |
| AED* | B |  | 350 | 477 | 3 | 0.0932 | B | B |
| Model 062M | C |  | 200 | 500 | 3 | 0.0977 | FLAT filter | B31f |

*Advanced Electron Density Phantom; NA = Not applicable
